# Phylotranscriptomics Shed Light on Intrageneric Relationships and Historical Biogeography of *Ceratozamia* (Cycadales)

**DOI:** 10.3390/plants12030478

**Published:** 2023-01-19

**Authors:** Sadaf Habib, Yiqing Gong, Shanshan Dong, Anders Lindstrom, Dennis William Stevenson, Hong Wu, Shouzhou Zhang

**Affiliations:** 1 College of Life Sciences, South China Agricultural University, Guangzhou 510642, China; 2 Key Laboratory of Southern Subtropical Plant Diversity, Fairy Lake Botanical Garden, Shenzhen and Chinese Academy of Sciences, Shenzhen 518004, China; 3 Global Biodiversity Conservancy 144/124 Moo 3, Soi Bua Thong, Bangsalae, Sattahip, Chonburi 20250, Thailand; 4 New York Botanical Garden, Bronx, NY 10458, USA

**Keywords:** Cycadales, *Ceratozamia*, single copy nuclear genes, transcriptomics, Trans-Mexican Volcanic Belt (TMVB)

## Abstract

*Ceratozamia* Brongn. is one of the species-rich genera of Cycadales comprising 38 species that are mainly distributed in Mexico, with a few species reported from neighboring regions. Phylogenetic relationships within the genus need detailed investigation based on extensive datasets and reliable systematic approaches. Therefore, we used 30 of the known 38 species to reconstruct the phylogeny based on transcriptome data of 3954 single-copy nuclear genes (SCGs) via coalescent and concatenated approaches and three comparative datasets (nt/nt12/aa). Based on all these methods, *Ceratozamia* is divided into six phylogenetic subclades within three major clades. There were a few discrepancies regarding phylogenetic position of some species within these subclades. Using these phylogenetic trees, biogeographic history and morphological diversity of the genus are explored. *Ceratozamia* originated from ancestors in southern Mexico since the mid-Miocene. There is a distinct distribution pattern of species through the Trans-Mexican Volcanic Belt (TMVB), that act as a barrier for the species dispersal at TMVB and its southern and northern part. Limited dispersal events occurred during the late Miocene, and maximum diversification happened during the Pliocene epoch. Our study provides a new insight into phylogenetic relationships, the origin and dispersal routes, and morphological diversity of the genus *Ceratozamia*. We also explain how past climatic changes affected the diversification of this Mesoamerica-native genus.

## 1. Introduction

Cycads, with 370 accepted species, are considered one of the oldest lineages of seed plants and are restricted to subtropical and tropical or warm temperate regions [1]. Cycads originated around 265 million years ago in the Permian period; however, extant cycads diversified as a result of rapid radiations in the last 11 to 20 Myr [2]. Four out of ten genera of cycads are distributed in the Neotropical region, i.e., *Ceratozamia* (38 species), *Dioon* (18 species), *Microcycas* (1 species) and *Zamia* (84 species) [1]. Among them, the former two genera have the greatest diversity in Mexico, with only few species described from neighboring regions of Guatemala and Belize and one outlier in each genus in Honduras [1,3,4].

Due to improved diagnostic methods and genetic data, knowledge about the systematics of Mexican cycads has been expanded greatly, with the number of representative species increasing in recent years. More than 20 cycad species have been described from Mexico in the past few years [3,5,6,7,8,9,10]. Increased understanding of species phylogenetic relationships and their biogeographic distribution in time and space is crucial for their conservation in the region because the majority of cycad species are considered threatened [11]. The primarily Mexican cycad genus, *Ceratozamia* Brongn. (Zamiaceae, Cycadales), contains ca. 38 species including some recently described species from Mexico [1,4,5,6]. Species of *Ceratozamia* have their greatest diversity in cloud forests, oak forests, and tropical rain forests on karstic rocks in Mexico and similarly with the three species that occur in Guatemala, Honduras, and Belize. The elevational range of the genus is from near sea level to ca. 2000 m [4].

*Ceratozamia* is monophyletic genus, sister to the genus *Stangeria* in a previous molecular data analysis [12]. In contrast, in other molecular analyses, *Ceratozamia* is resolved as the sister to a monophyletic clade composed of *Stangeria* and *Microcycas* + *Zamia* [2,13,14]. Morphological analyses have indicated that *Ceratozamia* is the sister to a clade comprising *Microcycas* + *Zamia* [15,16]. Several recent studies provide useful insights into the phylogenetic relationships within *Ceratozamia* using different molecular datasets [17,18]. *Ceratozamia* is either composed of two or three clades based on different datasets and comparative methods. Using three molecular markers (*nrITS, trnL-F, D-SCAR*), 16 species of *Ceratozamia* were divided into three main clades, with northern Mexico being resolved as an ancestral area for the genus [17]. A dataset of six molecular regions (*nrITS, rpoC1, matK, rbcL, psbK/I, atpF/H*) with an expanded coverage of 28 species revealed that *Ceratozamia* is composed of two major clades with low to moderately supported topologies [18]. Highly conserved sequences and low genetic diversity among *Ceratozamia* species has also been reported, using 31 species and *MatK* and *ITS* genetic regions [9]. This suggests that new molecular diagnostic characters such as single copy nuclear genes might be helpful to provide an improved understanding of infrageneric relationships as well as better resources for DNA barcoding for species identification in this genus and other cycad genera [13,19,20,21].

Protein-coding nuclear genes provide genetic information from both male and female inherence because they are biparentally inherited. Nuclear genes have been proven to be advantageous to resolve deep family/genus level relationships in all the major land plant groups [22,23,24,25,26,27]. These studies used either phylogenomics or phylotranscriptomics datasets with hundreds to thousands of genes, which demonstrate the great power of the nuclear genes toward addressing phylogenetic questions. A recent study demonstrated that extensive transcriptome data including thousands of single copy nuclear genes (SCGs) presented high resolution among closely related species in the cycad genus *Macrozamia* with relatively high evolutionary rates, while organellar protein-coding genes were less informative [19]. Therefore, we hypothesized that an extensive SCGs dataset would provide better resolved phylogenetic relationships within *Ceratozamia*, especially at the intrageneric level, and would further resolve the discrepancies found in earlier studies. Furthermore, a comparative dataset (nucleotide vs. amino acids) and analytical methods (concatenated vs. coalescent) would increase the confidence of the phylogenetic inferences among *Ceratozamia* species.

Within *Ceratozamia*, spatio-temporal diversification of the species has been studied recently [18]. According to this study, the cladogenesis events within the genus *Ceratozamia* started during the mid-Miocene (ca. 12 million years) and reached its maximum in the early Pleistocene (ca. 2 million years ago). Younger age estimates for the genus *Ceratozamia* have been provided indicating that the crown group was around 6 to 5.45 Ma, respectively, in those two previous reports [12,13]. Additionally, divergence times of *Ceratozamia* have been estimated in context of diversification ages of Cycadales using extensive phylogenomic analyses and expanded species coverage to 80% of the currently accepted *Ceratozamia* species, which had better node support at inter- or intra-generic levels [2,14]. Based on reliable sets of fossils records, the pattern of diversification in time and space among *Macrozamia* species was determined [19], which provided an explanation regarding species diversity in context of past climatic events. Thus, it is expected that these fossil calibrations and age estimations would also better explain the historical biogeography of the genus *Ceratozamia* and its current species distribution pattern. This in turn leads to a better understanding that spatial diversification has had an effect on environmental conditions and biogeographical history; thus, we explored the ancestral areas for the major clades and species within the genus *Ceratozamia*.

**Figure 1:**
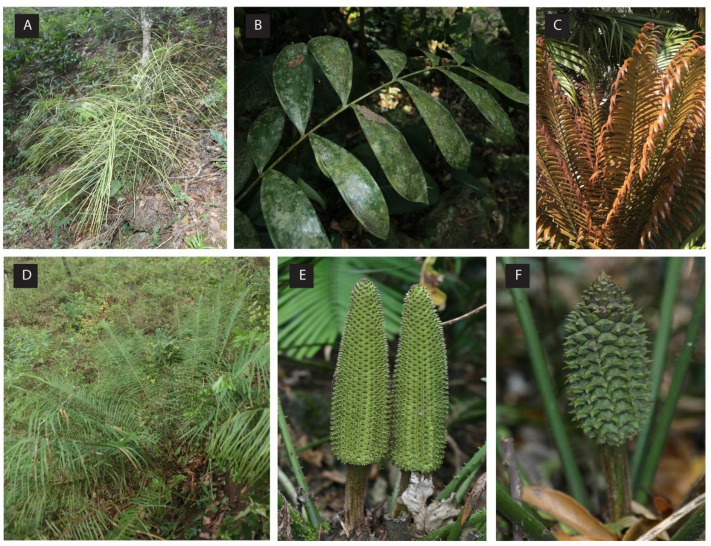
Species of *Ceratozamia* showing morphological variations. (**A**) *C. norstogii,* adapted for a shady, high-altitude, seasonal dry forest and displaying a twisted rachis with extremely narrow leaflets; (**B**) *C. hondurensis* adapted to a shady rainforest environment, and displaying broad leaflets; (**C**) *C. robusta* with distinct reddish-orange to dark maroon newly emerging leaflets; (**D**) *C. mirandae* from high-altitude open oak forest with narrow leaflets and a spreading leaf arrangement; (**E**,**F**) *C. tenuis* male vs. female cones displaying the distinct “horn-like” extensions that distinguish *Ceratozamia* from all other cycad genera. Photographs by Anders Lindstrom.

*Ceratozamia* are morphologically distinguishable in vegetative characters from closely related genera by the combination of two parallel adaxial canals along the rachis and entire leaflet margins. The presence of megasporophylls and microsporophylls with two distinct apical horns is an autapomorphy for the genus shown (Figure 1E,F) [4,28]. Species descriptions within the genus were traditionally based on morphological variations (Figure 1) such as leaf and leaflet morphology and the color and shape of strobilus indumentum [4,29,30]. In addition, some species diagnoses were based on geographical distribution and habitat differentiations, which is not a widely used approach and has limitations [4,31,32]. Several studies of the genus have evaluated the morphological variation among species populations with geographic and ecological affinities and, as a result, proposed the recognition of species complexes [6,7,18,33,34,35,36,37]. Additionally, morphological characters have been analyzed as quantitative and qualitative leaf morphological variations in the context of the species complexes [18].

Because previous efforts to establish species groups resulted in disparate results [9,17,18], an expanded sampling of species and markers is still needed to resolve infrageneric relationships within the species and species complexes. It has been proposed that in addition to traditionally used morphological characters of taxonomic utility from leaves, characters derived from reproductive structures should also be assessed to define relationships among species within the genus *Ceratozamia* [4]. Thus, an extensive exploration of morphological characters will provide further insight into their evolutionary history and ancestral states, especially the reproductive characters that have never been discussed in context of molecular phylogeny.

This study has four objectives: (1) Phylogenetic resolution of infrageneric relationships within the genus *Ceratozamia* using an extensive transcriptome dataset of SCGs, based on comparative dataset and analytical methods, (2) the historical biogeography and spatial distribution of this genus will be clarified based on the phylogenetic tree derived from the molecular evidence, (3) assessing the evolution of morphological characters in context of well-resolved phylogenetic tree, and (4) potential explanations regarding habitat variations impacting the current distribution pattern of *Ceratozamia*.

## 2. Results

A list of investigated species of the genus *Ceratozamia* and outgroup taxa with data submission information is given in Table 1. A total of 3954 SCGs were identified for reconstructing the phylogeny of *Ceratozamia*. The average alignment length is ~5539 bp, and missing data are from 0 to 27.7%. The aligned length of the concatenated dataset of all nucleotides (nt) was 5,052,760 bp, with 312,760 (6.18%) variable sites and 186,324 (3.68%) parsimony-informative sites. The aligned nucleotide dataset with first and second codon positions (nt12) was 3,369,107 bp long with 200,812 (5.9%) being variable and only 80,283 (2.38%) as the most parsimonious sites. The percentage of variable sites in the amino acid (aa) dataset was 21% (364,453) of the total aligned length (1,684,249 bp), which was the highest among all three datasets. However, only 4% (73,867 bp) of the sites were parsimony-informative. For divergence time estimation, 50 SCGs were selected that had a higher species coverage (>50%) and nucleotide divergence. The final alignment of this 50-gene dataset was around 56 kb.

### 2.1. Phylogenetic Trees

**Figure 2:**
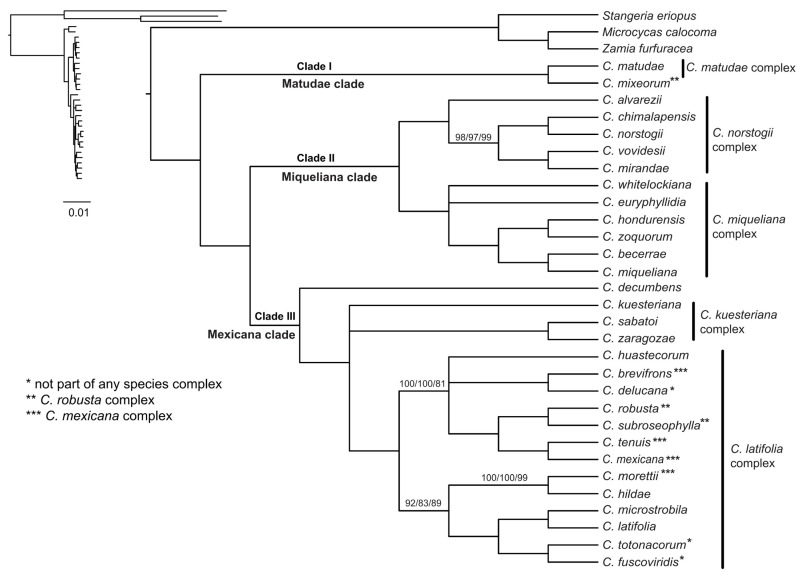
Congruent tree topology of *Ceratozamia* found by the maximum likelihood (ML) approach from the concatenated dataset of 3954 SCGs for 30 species. Numbers above branches are ML bootstrap (BS) from the nt/nt12/aa dataset, respectively. Only support values < 100% are shown. Branches with BS < 85 are collapsed. Seven species complexes adapted from Vovides [25,37] are marked in context of phylogenetic tree. A phylogram overview is shown in the upper left-hand corner with the scale bar indicating the average number of nucleotide substitutions per site.

**Figure 3:**
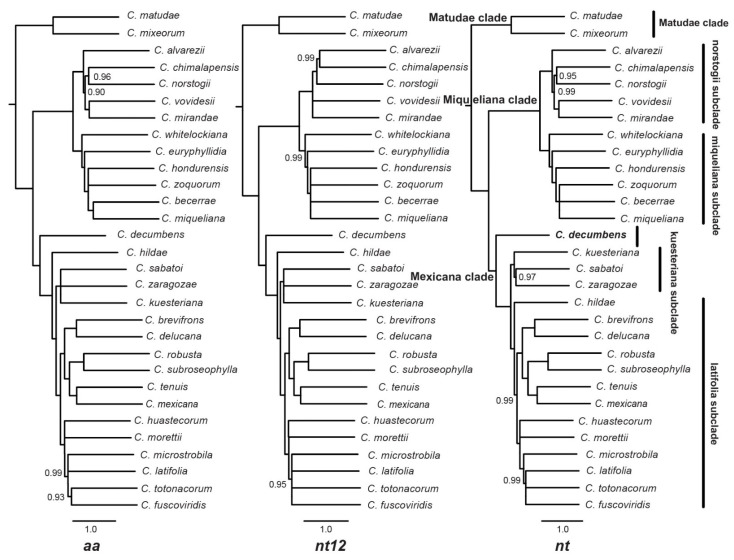
Maximum likelihood topology of the *Ceratozamia* species relationships inferred from the coalescence analyses of 3954 SCGs and the three comparative datasets (aa/nt12/nt). Posterior probability < 1.0 is given on the nodes. Branches with PP < 0.85 are collapsed. The six groups within the genus proposed in the current study are labeled.

The topologies produced from the ML tree of the concatenated dataset (nt, nt12, aa) were highly congruent, although with some conflicts of BS < 85 (Figure S1). When the topologies with BS < 85 were split into polytomies, we retrieved consistent results for the concatenated dataset (Figure 2). The phylogenetic inferences generated from the multi-species coalescent analyses in ASTRAL-III were mainly concordant among the three observed datasets (nt, nt12 and aa) and with the concatenated analyses (Figure S2). However, conflicts were retrieved for some internal nodes. The conflicting nodes with PP < 0.85 were split into polytomies (Figure 3), and only highly supported conflicts are discussed below.

Based on the concatenated ML tree with BS < 85 collapsed branches (ML-concat hereafter) and the multispecies MS tree with PP < 0.85 collapsed branches (MS-coal hereafter), *Ceratozamia* is divided into three major clades. The earliest diverging clade (clade I) consists of two species, i.e., *Ceratozamia mixeorum* and *C. matudae* (Figure 2 and Figure 3). The second clade (clade II) consists of 11 species and is further subdivided into two subclades. The interspecific relationships within these subclades were mostly consistent, but some discrepancies also occurred. The conflicting position of either *C. whitelockiana* or *C. euryphyllidia* as the earliest diverging species within a subclade of clade II is not resolved in the ML-concat tree (Figure 2). However, *C. whitelockiana* as the sister to all other species in this subclade is recovered in all three datasets (nt, nt12, aa) for MS-coal analyses (Figure 3 and Figure S2). For the second subclade within clade II, the position of *C. alvarezii* differed among the analyzed datasets. All ML-concat (BS = 100) and nt, aa datasets of the MS-coal tree (PP = 0.99) retrieved *C. alvarezii* as a sister to the other species within this subclade. However, in contrast, the nt12 dataset supported *C. alvarezii* as a sister to *C. chimalapensis* (PP = 0.99) at the inner most node within this subclade. The third major clade (clade III) consists of 17 species. *Ceratozamia decumbens* was a sister to all other species within this clade in all analyses. The other species within this major clade were divided into two subclades. *Ceratozamia sabatoi*, *C. zaragozae* and *C. kuesteriana* formed a weakly supported clade in the ML-concat datasets. Moreover, this subclade is maximally supported (PP = 1) in all the MS-coal datasets. The remaining 13 species were grouped into one subclade with several poor to highly supported relationships. Conflicting topologies were retrieved for the phylogenetic positions of *C. hildae* and *C. huastecorum*. The fairly supported alternative phylogenetic position of *C. hildae* was retrieved among the two different analytical methods (ML-concat and MS-coal) and in the different dataset of nt, nt12, aa within the MS-coal analyses (Figure 3 and Figure S2). In the case of *C. huastecorum*, the conflict occurred between the phylogenetic inferences from the ML-concat and MS-coal analyses. However, neither of these two positions retrieved well supported relationships with a BS > 75 and a PP > 0.85 with their immediate sister groups.

The quartet score calculated by ASTRAL (Figure S3) was higher in the main topology (q1) as compared to the alternative ones (q2, q3) for the three major clades (clade I: q1 = 0.98, q2 = 0.01, q3 = 0.01; clade II: q1 = 0.48, q2 = 0.3, q3 = 0.22; clade III: q1 = 0.4, q2 = 0.34, q3 = 0.25) and for the subclades within clade II (q1 = 0.64, q2 = 0.18, q3 = 0.19). In contrast, the phylogenetic inferences within clade III were low (>40%) at most of the internal nodes. All the species with conflicting positions among the ML-concat and the MS-coal approaches or among the nt, nt12, aa datasets also were revealed to have low quartet support for alternative topologies.

### 2.2. Biogeography and Habitat Description

**Figure 4:**
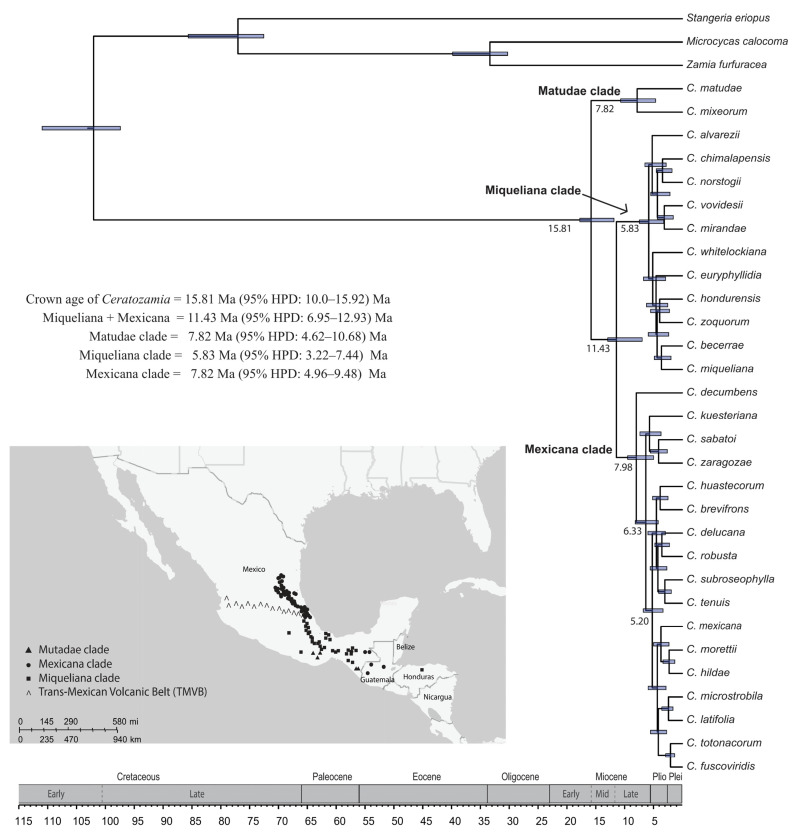
Chronogram for *Ceratozamia* reconstructed based on the combined data matrix of 50 SCGs constrained to the ML tree of the concatenated dataset with all codon positions included (nt dataset). Node bar on each node represents the minimum and maximum ranges of dates from both low and high calibration. The map shows the geographical extents of the three major clades among the three distinct areas of its distribution in Mexico and its adjacent regions.

As the ML tree topologies were mostly consistent among the three major clades and subclades included within them, we described the results from the constrained 50-gene ML tree to the topologies derived from only the ML-concat analyses. This 50-gene ML tree was then used for subsequent diversification analyses (Figure 4). The crown group of *Ceratozamia* was estimated to be 15.81 Ma (95% HPD: 10–15.92 Ma) during the mid-Miocene (Figure 4). The split between clade II and III occurred at 11.43 Ma (95% HPD: 6.95–12.93 Ma) during the late Miocene. Speciation within clade I, II and III started within the same sub-epoch of Miocene around 7.8 Ma (95% HPD: 4.62–10.68 Ma), 5.82 Ma (95% HPD: 3.22–7.44) and 7.98 Ma (95% HPD: 4.96–9.48 Ma), respectively (Figure 4). However, the major expansions of species occurred during the Pliocene (18 species) with fewer in the Pleistocene (6 species) (Figure 4). 

**Figure 5:**
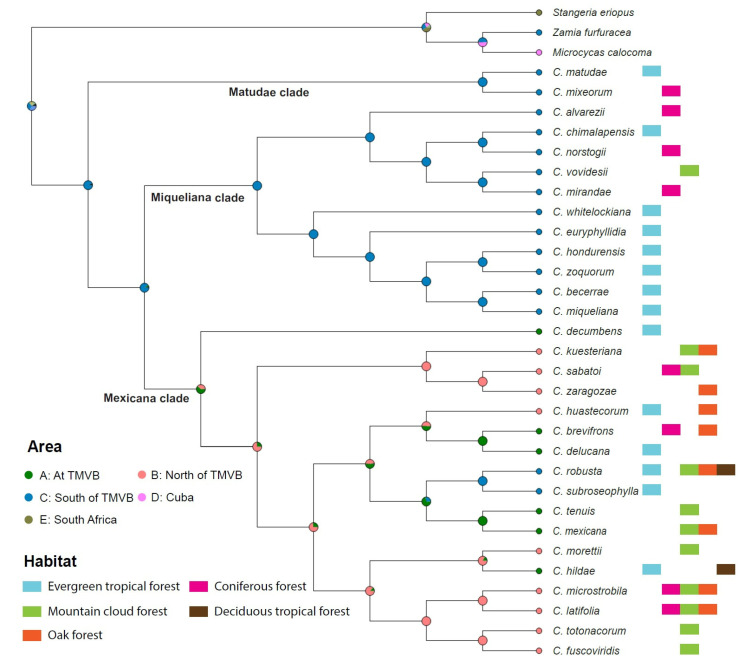
Ancestral area reconstruction for the genus *Ceratozamia* based on the combined data matrix of 50 single-copy nuclear genes constrained to the ML tree of the concatenated dataset with all codon positions included (nt dataset). The geographical extents of the three areas with reference to the Trans-Mexican Volcanic Belt (TMVB) as (A) at TMVB, (B) north of TMVB, and (C) south of TMVB considered in the analysis. The colored circles on the nodes show the most likely ancestral areas reconstructed by BioGeoBEARS. Current distributions are indicated at the branch tips. Habitat diversity for each *Ceratozamia* species is given to the right of the species name.

The species within *Ceratozamia* are clearly divided into major clades based on their biogeographical distribution in the south and north at the Trans-Mexican Volcanic Belt (TMVB) in Mexico (Figure 5). The region at the south of the TMVB was found to be the ancestral area (Area C: 93% probability) for the entire genus (Table 2). The species of clades I and II are distributed in the south of the TMVB. The dispersal of species from southern to northern Mexico occurred though the TMVB, which is the ancestral area (Area B) of clade III with a higher probability (Area A: 65%) than the northern region (Area B: 33%) (Figure 5, Table 2). After reaching the north, the migration back to the TMVB occurred four times in the last 2–4 Ma and once toward the south through the TMVB.

The highest abundance of *Ceratozamia* species occurs in evergreen tropical forests, which is the most common habitat type in the south of the TMVB (Figure 5). Next in abundance is the “mountain cloud forest”, and the species were confined to the TMVB and its northern part in Mexico. Species present in coniferous forests have a wide range of distribution from the south to the north. Some species have a broader habitat range such as *C. robusta*, grown at all habitat ranges except for coniferous forests.

### 2.3. Character Evolution

**Figure 6:**
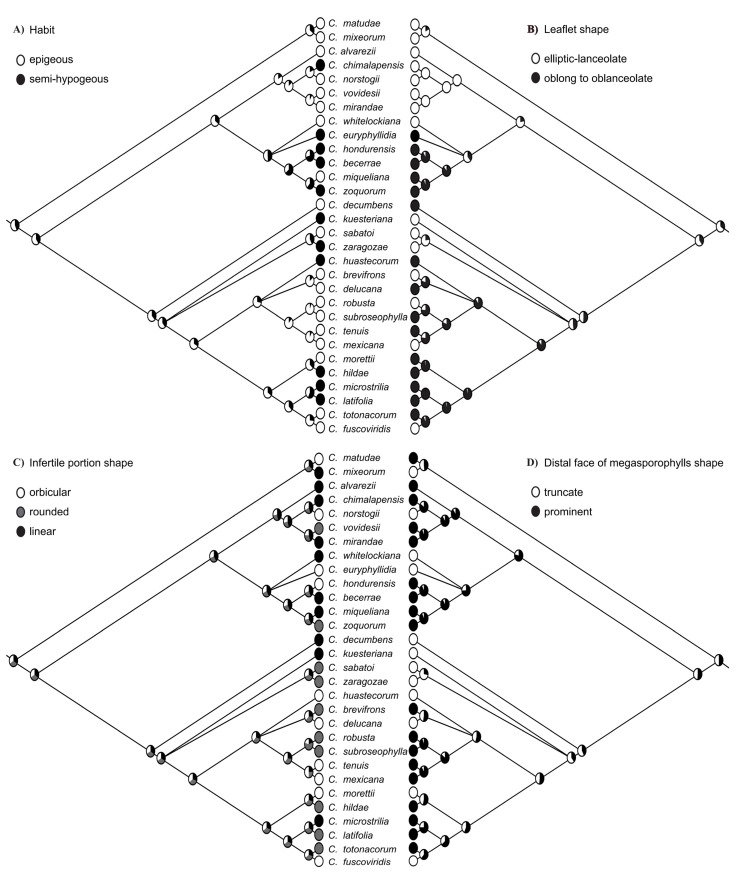
Optimization of key morphological characters based on the Mk1 model implemented in Mesquite. (**A**) Habit (character 1), (**B**) leaflet shape (character 2), (**C**) infertile portion shape (character 8), and (**D**) distal face of megasporophyll (character 11) inferred from the ML tree of the concatenated dataset with all codon positions included (nt dataset) and with poorly supported branches (BS < 85) collapsed.

Likelihood inferences of character states indicated that all 12 selected morphological characters (Table 3) are homoplastic in *Ceratozamia*. Among these, only a few such as “habit”, “leaflet shape”, “infertile portion shape” and “distal face of megasporophylls shape” provide distinctions to separate clades or subclades (Figure 6). Mapping of the alternating states for four characters, i.e., “leaflet direction”, “microsporophylls shape”, “angle between horns of megasporophylls”, and “sarcotesta color” appears to be extremely complex (Figure S4). Ancestral states for all of above-mentioned characters were uncertain for the whole genus, as none of the recorded states attain significant probabilities (>75%). On the other hand, “leaflet texture”, “formation of prickles”, “angle between horns of microsporophylls”, and “fertile portion shape” showed less variations (Figure S5).

## 3. Discussion

### 3.1. Phylogeny Based on Extensive Transcriptome Data

Increasing the number of genes provided high branch support for relationships within *Ceratozamia*, which were much lower, and not the optimal estimates, with fewer loci in previous studies [9,17,18,31]. Relationships among the three major clades were robust. There was a general agreement among trees generated from the comparative methods (coalescent vs. concatenated) and the datasets (nt, nt12, aa). The topological discordance between gene trees revealed a gene tree heterogeneity at shallow internal branches, especially for the species within clade III. The nearly identical quartet frequencies for alternative topologies in our datasets suggested that incomplete lineage sorting (ILS) is likely to impede phylogenetic resolution, which is evident from the very short branches for these topologies. Bootstrap support from the nucleotide dataset did not show an increase in branch support, compared to amino acid data, that is generally observed in cycads [19].

In a recent study, *Ceratozamia* was divided into two major clades: “Miqueliana” and “Mexicana”. *C. matudae* and *C. mixeorum* were distantly placed into these two clades, respectively [18]. In contrast, the current study placed these two taxa in clade I as a sister to all remaining taxa within the genus, i.e., Miqueliana clade (clade II) and Mexicana clade (clade III); thus, supporting a previous hypothesis based on a limited taxon sampling and dataset [17]. Our study provides reliable phylogenetic inferences from different comparative methods and datasets for the genus, based on 3954 SCGs including 30/38 species of *Ceratozamia*.

### 3.2. Evolution of Species Complexes in Context of Phylogeny

Twenty-one species of *Ceratozamia* were divided into seven species complexes, based upon data from their geographic distribution, habitat preferences and gross morphology [30]. Later studies added several species to these species complexes [6,7,9,35,38]. Species of the *Ceratozamia miqueliana* complex and the *Ceratozamia norstogii* complex were grouped as two monophyletic subclades within the Miqueliana clade. The *Ceratozamia miqueliana* complex was described based on species distribution in the south and southeast of the TMVB, with wider leaves and leaflets [30,38,39]. These species are only found in evergreen tropical rain forest and are not generally found at elevations greater than 1000 m [40]. Previously, *C. whitelockiana* was suggested to be closely allied to *C. miqueliana* [38,41] based on shared cone and leaf characters. However, this species has also been considered to be part of the *Ceratozamia robusta* complex [30]. Our phylogenetic reconstruction affirmed the placement of *C. whitelockiana* within the *Ceratozamia miqueliana* complex in all the analyses and comparative methods.

The *Ceratozamia norstogii* complex sensu Vovides [29] includes *C. norstogii*, *C. alvarezii* and *C. mirandae*. Based on the current well-supported phylogeny, we recognize the inclusion of *C. chimalapensis* and *C. vovidesii* within this complex. This is further supported because these species share leaf and cone morphological features with the species of the *Ceratozamia norstogii* complex [42,43]. *Cerastozamia chimalapensis* and *C. vovidesii* occur in evergreen tropical forests and mountain cloud forests, respectively. The other three species, *C. norstogii*, *C. alvarezii*, and *C. mirandae*, are restricted to coniferous forests. *Ceratozamia matudae* also shares morphological characters with the species of the *C. norstogii* complex [42]. However, current distant phylogenetic placement (Figure 4) of the species in the *matudae* clade shows its distinction from this complex. Previous studies also considered *C. matudae* as a separate species complex from the extreme south of Chiapas into Guatemala [29].

Within the Mexicana clade, *C. decumbens* is a sister to two subclades (Figure 2). One of these is the kuesteriana subclade with the three species (C. *kuesteriana* sister to *C. zaragozae + C. sabatoi*) belonging to the *Ceratozamia kuesteriana* complex, which share narrow lanceolate to linear leaflets and close geological proximity in northeastern Mexico [29]. The monophyly of this complex was highly supported in the ML-coal analyses. The second subclade is the *latifolia* subclade, which is a sister to the *kuesteriana* subclade (Figure 2). This subclade also includes species of the *Ceratozamia mexicana* complex and *C. robusta* complex. None of these two species complexes formed the monophyletic group. The circumscription of the *C. robusta* complex has remained problematic historically and was partially disentangled in some taxonomic studies [7,33]. All other taxa, which never included any of the other species complexes within *Ceratozamia*, were also within this clade (Figure 2). Among the seven described species complexes within *Ceratozamia* [29,33,36,37,44], our study confirmed the monophylly of only three species complexes, i.e., *C*. *norstogii* complex, *C. miqueliana* complex and *C. kuesteriana* complex, whereas all others are either para- or polyphyletic. In light of the current evidence, we suggest the following six infrageneric groups within three major clades of the genus *Ceratozamia*: (1) Matudae clade (I), (2) norstogii subclade (Miqueliana clade, II), (3) miqueliana subclade (Miqueliana clade, II), (4) *C. decumbens* (Mexicana clade, III), (5) kuesteriana subclade (Mexicana clade, III), and (6) latifolia subclade (Mexicana clade, III). The broader circumscription of the latifolia subclade as a single group is suggested because the phylogenetic position of species within this subclade showed several media to poorly supported relationships (Figure 3). Furthermore, newly described species within this group are morphologically similar to the species from different species complexes placed within this monophyletic subgroup. For instance, *C. totonacorum* shares morphological affinities with *C. mexicana, C. tenuis, C. delucana, C. fuscoviridis* and *C. morettii,* all of which are placed within the latifolia subclade [35]. Similarly, *Ceratozamia brevifrons* is morphologically similar to *C. robusta* (of the *C. robusta* complex) and *C. mexicana* (of the *C. mexicana* complex) [45].

### 3.3. Age Estimations and Biogeographic Associations

The divergence between *Ceratozamia* and its immediate sister group (a clade consisting of *Microcycas*, *Zamia* and *Stangeria*) occurred during the early Cretaceous around 102 Ma [2]. Most of the diversity of the genus *Ceratozamia* is in Southern Mexico with 21 out of the 38 species occurring there. Previously, phylogeographic investigation of the genus reported that *Ceratozamia* diverged during the mid-Miocene (ca. 12 Ma), and major cladogenesis events started within the genus during the Pliocene (ca. 5 Ma), reaching the maximum rate during the Pleistocene (2.5 Ma). Furthermore, it was believed that diversification of the “Mexicana clade” started earlier than the “Miqueliana clade”, and there was a free exchange of species among the three biogeographical areas of *Ceratozamia* distribution [18,32]. In contrast to the above cited study, our study reported three major clades within the genus with slightly older age estimates of crown age dated to the mid-Miocene (ca. 16 Ma). The highest species divergence occurred during the Pliocene, and the speciation rates have been slowing down toward the present, as only six speciation events occurred during the Pleistocene. It is affirmed that the *Ceratozamia* species originated from an ancestor from the south of TMVB, with a secondary colonization at TMVB and its northern part. There were multiple migrations between the north and at the TMVB; however, species dispersal back to the south from the north or at the TMVB was limited. The earlier diverging *C. decumbens* inhabited the TMVB in mountain tropical forests during the Late Miocene, at ~8 Ma. Its presence as a sister to the taxa situated at and north of the TMVB appeared to be the product of a recent vicariance event [31]. Our divergence age estimates revealed the rapid diversification among *Ceratozamia* species in a short time frame (at 11.43 Ma) and fast overland dispersal between, at, or north of the TMVB, and ancestral southern Mexico taxa. The spatio-temporal diversification pattern of *Ceratozamia* is shaped by fluctuating climatic factors, which arose due to geological events. The vegetation history of southern Mexico to Belize indicated that these regions retained relict floral elements of great age [46,47,48]. Refuges of flora and fauna of ancient times (40,000 years) have been postulated in southern Mexico, which were apparently absent at the Neovolcanic Mountain Range and its northern region [49,50]. The last warm epoch was present in the late and mid-Miocene followed by consecutive cooling events [51]. Palynomorph studies in Mexico mentioned fluctuating climate that became increasingly temperate with semiarid regions intermixed with sub-humid regions, and increased climatic seasonality were responsible for the expansion and divergence of tropical deciduous forests [52,53,54]. Further evidence drawn from lithological studies indicated that the mountainous regions became more arid while the low-lying areas received maximum precipitation [55]. This evidence supports the dispersal time and genetic divergence of a closely related taxa of *Ceratozamia* into present-day Honduras and bordering eastern Guatemala and western Belize.

### 3.4. Evolution of Morphological Characters

The evolution of morphological characters is extremely complex in *Ceratozamia.* Some important diagnostic characters showed homoplasy in character states across the phylogenetic tree. Only four (characters # 1, 2, 8, 11) out of the 12 characters provide some evidence to define one or more major clade, whereas others exhibit considerable convergent evolution (Figure 6, Figures S4 and S5). These four characters are habit, leaflet shape, infertile portion shape and the shape of the distal face of megasporophylls. Most of the species within *Ceratozamia* have an “epigeous” habit; however, “semi-hypogeous” stems are dominant in the miqueliana and kuesteriana subclades. In addition, this character state has evolved once in the norstogii subclade and thrice in the latifolia subclade (Figure 6A). The ancestral state of leaflet shape among six groups within *Ceratozamia* is uncertain, and the probability of the two alternating states is low (elliptic–lanceolate = 55%, vs. oblong to oblanceolate = 45%). The leaves of the Matudae clade and the norstogii and kuesteriana subclades are elliptic–lanceolate, and this state also arose once in the miqueliana subclade and four times in the latifolia subclade (Figure 6B). Leaflet shape in *Ceratozamia* appears to be primarily related to their habitat types. With respect to reproductive characters, “linear” infertile portion shape is more common in the norstogii and miqueliana subclade, and it independently evolved once in the matudae and kuesteriana subclades and in *C. decumbens*. Most of the species in these subclades either have “orbicular” or “rounded” infertile portions (Figure 6C). The distal face of the megasporophyll is “prominent” in most of the species in the latifolia, norstogii and miqueliana subclades, whereas *C. decumbens* and the kuesteriana subclade have a “truncated” distal face of the megasporophylls (Figure 6D).

In other cycad genera, as in the genus *Ceratozamia*, both qualitative and quantitative variation of vegetative characters derived mainly from leaf morphology are widely used for species identification, e.g., [4,9,32,56,57,58,59,60]. This is the first study to evaluate reproductive morphological characters in addition to vegetative characters in broad phylogenetic context within *Ceratozamia*. We confirmed the extremely complex pattern of vegetative as well as reproductive characters in the genus because they are uninformative either because of little variation or because of high polymorphism within and between populations. Nevertheless, there are some morphological distinctions to represent the phylogenetic groups within the genus (Figure 4). In light of the above-mentioned issues, we affirmed the fact that the infrageneric phylogenetic groups within the genus do not have morphological distinction, but rather other factors such as biogeographical distinction, as in other cycads genera [3,19,61].

## 4. Materials and Methods

### 4.1. Sampling, Transcriptome Data Processing and Phylogenomic Analyses

A comprehensive transcriptomes analysis of data of 339 cycad species was conducted in our recent research to explore the early evolution of seed plants along with whole genome sequencing of *Cycas panzhihuaensis* [2]. We retrieved the transcriptome data of 30 *Ceratozamia* species from this study to investigate the phylogenetic relationships within *Ceratozamia*. Additionally, three species (*Microcycas calocoma*, *Stangeria eriopus* and *Zamia furfuracea*) were selected from closely related genera as an outgroup. RNA-seq data were submitted to the National Center for Biotechnology Information (NCBI) under the BioProject ID PRJNA922610. All the species included in our study were collected from the Nong Nooch Tropical Botanical Garden, Pattaya, Thailand (NNTBG), and voucher specimens were submitted at The Singapore Botanic Gardens Herbarium (SING), Singapore and Natural History Museum (W), Vienna Austria (Table 1).

Data processing mainly followed the recent phylotranscriptomic study of the cycad genus *Macrozamia* [19]. The clean reads were de novo assembled using the Trinity pipeline [62]. TransDecoder (https://github.com/TransDecoder, accessed on 12 March 2022) was used for predicting coding regions of each putative transcript and annotation for assembled transcriptomes. In the next step, orthologs were recognized from annotated transcripts using OrthoFinder v2.5.4 [63]. Then, single-copy genes were selected for phylogenetic reconstruction using Software KinFin v1.1 [64] with default parameters. Gene families containing at least 24 in-group species (>80%) were selected, which then obtained 3954 candidate homologous clusters. Gene dataset was filtered using a local version of program TranslatorX vLocal.pl [65] in the following four steps: (1) use the standard genetic code to translate the nucleotide sequences into amino acids sequences; (2) align these peptide sequences of each putative SCG with MAFFT v.7.505 [66]; (3) further trim the amino acid sequences that exclude ambiguous portions using Gblocks [67]; (4) convert the alignments into the corresponding nucleotide alignments.

For phylogenomic analysis, we used two comparative methods for the SCG dataset, i.e., coalescence- and concatenation-based methods. For the coalescent-based method, single-gene maximum likelihood (ML) trees were reconstructed in IQ-TREE 2 [68], with ultra-fast bootstrap analysis of 100 replicates. All those trees were used as input in ASTRAL-III [69] for species tree inference. To calculate quartet support (q) for each branch including the main topology and the first and second alternatives, option “-t 8” was used in ASTRAL-III. For the concatenation method, genes of all the datasets were concatenated using SeqKit [70], and then, we applied the ML method as implemented in IQ-TREE 2 [68]. The best fitting model is selected by ModelFinder [71], in IQ-TREE 2. Finally, we obtained phylogenetic trees for three datasets, i.e., nucleotide data including all codon positions (nt), nucleotide data from first and second codon position (nt 12) and amino acid alignments (aa) from each of the coalescent and concatenated analyses. Due to the possibility of incongruence among trees from comparative datasets and analyses, the poorly supported branches with <85% support were collapsed using TreeCollapserCL4 [72].

### 4.2. Molecular Dating, Ancestral Area Reconstruction, and Habitat Assessment

The dating analysis was performed in the following steps: (1) (1) 50 genes were manually selected to produce a 50-gene ML tree for dating analyses with the highest species coverage and sufficient variable sites. (2) Those 50-genes were aligned using MAFFT v.7.505 [66], and the ML trees were reconstructed by RAxML v.8.2.12 [73] under the GTR + G model. (3) Keeping in view the possible conflict among tree topologies from coalescent and concatenated datasets, the tree topologies were constrained initially to the nt tree that resulted from both analyses. (4) We used TreePL [74] to date the divergence times of species within the genus *Ceratozamia* with the 100 generated from the RAxML bootstrap replicates. For this analysis, we constrained both the root (min: 76.29, max: 110.96) and the crown ages of the genus (min: 10.00, max: 16.29) by using the time estimations found in MCMCTree dating analysis of our previous study of cycads diversification [2]. (5) The maximum clade credibility (MCC) trees were then constructed with median ages and 95% highest posterior density (HPD) intervals on nodes using TreeAnnotator v2.6.2 [75]. We used FigTree v.1.4.4 (https://tree.bio.ed.ac.uk/software/figtree; accessed on 2 May 2022) to visualize the final chronogram.

The final chronogram was also used as input to search and execute the best model in the R package “BioGeoBEARS” [76], which was DIVALIKE + J. Distribution data for *Ceratozamia* species were mainly derived from herbarium records given in [4] and online at the Global Biodiversity Information Facility (GBIF, http://www.gbif.org/; accessed on 8 May 2022). The Trans-Mexican Volcanic Belt (TMVB) in Mexico is considered an important barrier shaping the spatial patterns and geographic diversification of *Ceratozamia* [18,31]. Following the approach used in [18], three spatial regions were assigned to 30 *Ceratozamia* species based on their distribution patterns with respect to TMVB, i.e., Area A: at TMVB, Area B: north of TMVB, Area C: south of TMVB. We allowed the inferred ancestors to occupy a maximum of two areas (sampled tips occupy only one area).

We assessed the distribution pattern of vegetations types on the phylogenetic tree (Figure 5). There are five vegetation ranges where the *Ceratozamia* species are distributed: (1) evergreen tropical forest, (2) coniferous forest, (3) mountain cloud forest, (4) deciduous tropical forest, and (5) oak forests. The data of vegetation type were recorded from [4,16].

### 4.3. Morphological Characters State Mapping

Initially, we designed a character matrix including around 20 morphological characters from stems, leaves, pollen and ovulate strobili and seeds. We removed characters if there was little variation among species such as species autapomorphies. For example, all but one species had “recurved” distal face of microsporophylls in *Ceratozamia*; thus, this character was removed. The final data analyses included 12 morphological characters (Table 2). The character states and coding mainly followed the most recent monograph of *Ceratozamia* [4]. The ML tree from the concatenated dataset was used as input to infer the character states using the ML approach as implemented in Mesquite v. 3.70 [77]. The Markov k-state one-parameter model of evolution for discrete unordered characters was used [78]. Outgroup species were pruned from the tree because most morphological characters evaluated here were unknown/not present in those taxa.

## 5. Conclusions

Due to variable molecular markers and analytical methods investigated earlier, phylogenetic relationships within *Ceratozamia* have been a contentious issue. Based on the coalescent and concatenated approaches utilizing 3954 SCGs, our study yielded a reliable phylogenetic tree of *Ceratozamia*. It comprises six major branches/subclades with three major clades. The main cause of conflicts observed among the gene trees is suggested to be a rapid evolutionary divergence and ILS, which is evident from the low quartet support values among the gene trees. Molecular dating and diversification analysis based on the 50 concatenated SCGs suggests that *Ceratozamia* originated around 15.81 Mya, during the mid-Miocene in southern Mexico, which is considered to be a biodiversity hotspot of neotropical cycads. Our study showed that the major groups within the genus are consistent with their geographical distribution and sometimes with morphological variations. On the basis of combined molecular, biogeographical and morphological evidence, the six monophyletic subclades could be proposed as distinct groups in a formal infrageneric classification of the genus.

We concluded that global climate changes have been intensified now, which predict the extinction of the cycad tree of life, as a considerable decline in speciation occurred toward the present [19,79]. Therefore, extensive studies are required to understand the evolutionary fate of cycad species in their respective biodiversity hotspots, which would provide future guidelines for conservation and management practices.

## Figures and Tables

**Table 1:** List of *Ceratozamia* species and the three outgroup taxa included in this study for phylogenetic analyses.

No.	Sample ID *	Taxon Names	Raw Data (Gb)	Clean Reads (Gb) *	Accession No. *	Herbarium Code
1	SAMN32670532	*Ceratozamia alvarezii*	6	3.32	SRR23044200	SING
2	SAMN32670533	*Ceratozamia becerrae*	6	3.32	SRR23044199	SING
3	SAMN32670534	*Ceratozamia brevifrons*	6	3.35	SRR23044188	W
4	SAMN32670535	*Ceratozamia chimalapensis*	6	3.26	SRR23044177	SING
5	SAMN32670536	*Ceratozamia decumbens*	6	3.29	SRR23044173	W
6	SAMN32670537	*Ceratozamia delucana*	6	3.47	SRR23044172	SING
7	SAMN32670538	*Ceratozamia euryphyllidia*	6	3.21	SRR23044171	SING
8	SAMN32670539	*Ceratozamia fuscoviridis*	6	2.94	SRR23044170	W
9	SAMN32670540	*Ceratozamia hildae*	12	6.49	SRR23044169	W
10	SAMN32670541	*Ceratozamia hondurensis*	6	3.40	SRR23044168	SING
11	SAMN32670542	*Ceratozamia huastecorum*	6	3.38	SRR23044198	SING
12	SAMN32670543	*Ceratozamia kuesteriana*	6	3.09	SRR23044197	W
13	SAMN32670544	*Ceratozamia latifolia*	6	3.36	SRR23044196	W
14	SAMN32670545	*Ceratozamia matudae*	6	3.31	SRR23044195	SING
15	SAMN32670546	*Ceratozamia mexicana*	6	3.35	SRR23044194	SING
16	SAMN32670547	*Ceratozamia microstrobila*	6	3.38	SRR23044193	W
17	SAMN32670548	*Ceratozamia miqueliana*	6	3.27	SRR23044192	W
18	SAMN32670549	*Ceratozamia mirandae*	6	3.35	SRR23044191	W
19	SAMN32670550	*Ceratozamia mixeorum*	6	3.46	SRR23044190	W
20	SAMN32670551	*Ceratozamia morettii*	6	3.44	SRR23044189	SING
21	SAMN32670552	*Ceratozamia norstogii*	6	3.41	SRR23044187	W
22	SAMN32670553	*Ceratozamia robusta*	6	3.31	SRR23044186	SING
23	SAMN32670554	*Ceratozamia sabatoi*	6	3.33	SRR23044185	W
24	SAMN32670555	*Ceratozamia subroseophylla*	6	3.38	SRR23044184	W
25	SAMN32670556	*Ceratozamia tenuis*	6	3.91	SRR23044183	SING
26	SAMN32670557	*Ceratozamia totonacorum*	6	3.34	SRR23044182	W
27	SAMN32670558	*Ceratozamia vovidesii*	6	3.23	SRR23044181	W
28	SAMN32670559	*Ceratozamia whitelockiana*	6	3.4	SRR23044180	SING
29	SAMN32670560	*Ceratozamia zaragozae*	6	3.35	SRR23044179	W
30	SAMN32670561	*Ceratozamia zoquorum*	6	3.43	SRR23044178	W
31	SAMN32670562	*Microcycas calocoma*	12	6.6	SRR23044176	W
32	SAMN32670563	*Stangeria eriopus*	12	6.85	SRR23044175	W
33	SAMN32670564	*Zamia furfuracea*	12	7.79	SRR23044174	W

* NCBI database.

**Table 2:** Ancestral area probability (% age) based on Figure 4 for each of the key nodes of the genus *Ceratozamia*.

	Area A: At TMVB	Area B: North of TMVB	Area C: South of TMVB
**Whole genus**	2.67	1.61	94.22
Matudae clade	0	0	100
**Miqueliana + Mexicana**	5.94	3.5	90.23
**Miqueliana clade**	0	0	100
norstogii subclade	0	0	100
miqueliana subclade	0	0	100
**Mexicana clade**	61.57	37.24	>1
kuesteriana subclade	100	0	0
latifolia subclade	72.44	26.45	1.09

**Table 3:** List of morphological characters traced on the phylogenetic tree of the genus *Ceratozamia*.

	No.	Characters	States		
			0	1	2
Stem	1	Habit	Epigeous	semi-hypogeous	
Leaves	2	Leaflet shape	elliptic-lanceolate	oblong to oblanceolate	obovate
	3	Leaflet direction	Descending	ascending	
	4	Leaflet texture	Coriaceous	papyraceous	membranaceous
	5	Formation of prickles (Petiole)	Robust	thin	
Microsporangiate strobili	6	Microsporophyll shape	Discoid	obconic	elliptic
	7	Angle between horns of microsporophyll	Obtuse	acute	right
	8	Infertile portion shape	Orbicular	rounded	linear
	9	Fertile portion shape	Lobate	deeply lobate	
Megasporangiate strobili	10	Angle between horns of megasporophylls	Obtuse	acute	right
	11	Distal face of megasporophylls shape	Truncate	prominent	
Seed	12	Sarcotesta color	light-brown	whitish-yellow	whitish-red

## Data Availability

The clean reads data are available at NCBI database under the Bioproject ID PRJNA922610, and SRA accession number of SRR23044168—SRR23044200.

## Supplementary Materials

The following supporting information can be downloaded at: https://www.mdpi.com/article/10.3390/plants12030478/s1. Figure S1:Phylogenetic relationships within Ceratozamia based on con-catenated dataset of (A) nt, (B) nt12, (C) aa, from 3954 Single Copy Nuclear genes (SCGs); Figure S2: Phylogenetic relationships within Ceratozamia based on coalescent dataset of (A) nt, (B) nt12, (C) aa, from 3954 Single Copy Nuclear genes (SCGs); Figure S3: Species trees of the genus Ceratozamia generated in ASTRAL-III for nucleotide dataset with 1st and 2nd codon position (nt12). Support values from quartet analysis are mentioned for each node. The low support values of the con-sensus highlight the amount of incongruence among the gene trees; Figure S4: Optimization of morphological characters with complex pattern, based on Mk1 model implemented in Mesquite. (A) Leaflet direction (character 3), (B) Microsporophyll shape (character 6), (C) Angle between horns of megasporophylls (character 10), and (D) Sarcotesta color (character 12) inferred to ML tree of concatenated dataset with all codon position included (nt dataset), with poorly supported branches (BS < 85) collapsed; Figure S5: Optimization of less variable morphological characters, based on Mk1 model implemented in Mesquite. (A) Leaflet texture (character 4), (B) Leaflet di-rection (character 5), (C) Angle between horns of microsporophyll (character 7), and (D) Fertile portion shape (character 9) inferred to ML tree of concatenated dataset with all codon position included (nt dataset), with poorly supported branches (BS < 85) collapsed.
