# Supplementary material for: Phylotranscriptomics Shed Light on Intrageneric Relationships and Historical Biogeography of Ceratozamia (Cycadales)
Source: Plants (Basel). 2023 Jan 19;12(3):478. doi: 10.3390/plants12030478 (PMC9921377; doi:10.3390/plants12030478)
Supplement: Supplementary file 1 [file plants-12-00478-s001.zip › plants-2117219-supplementary.pdf]

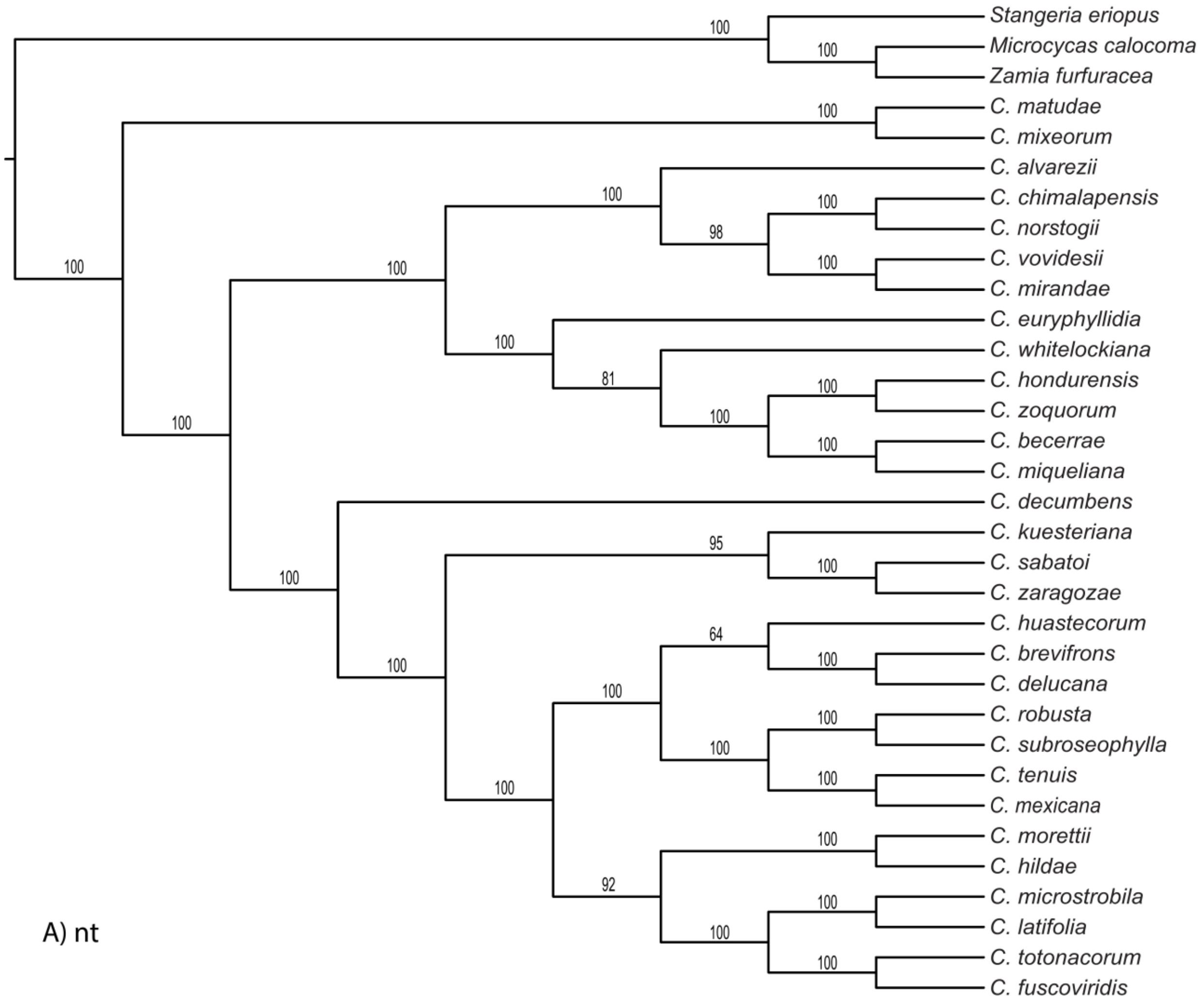
Stangeria eriopus
Microcycas calocoma
Zamia furfuracea
C. matudae
C. mixeorum
C. alvarezii
C. chimalapensis
C. norstogii
C. vovidesii
C. mirandae
C. euryphyllidia
C. whitelockiana
C. hondurensis
C. zoquorum
C. becerrae
C. miqueliana
C. decumbens
C. kuesteriana
C. sabatoi
C. zaragozae
C. huastecorum
C. brevifrons
C. delucana
C. robusta
C. subroseophylla
C. tenuis
C. mexicana
C. morettii
C. hildae
C. microstrobila
C. latifolia
C. totonacorum
C. fuscoviridis
100
98
95
92
81
64

A) nt

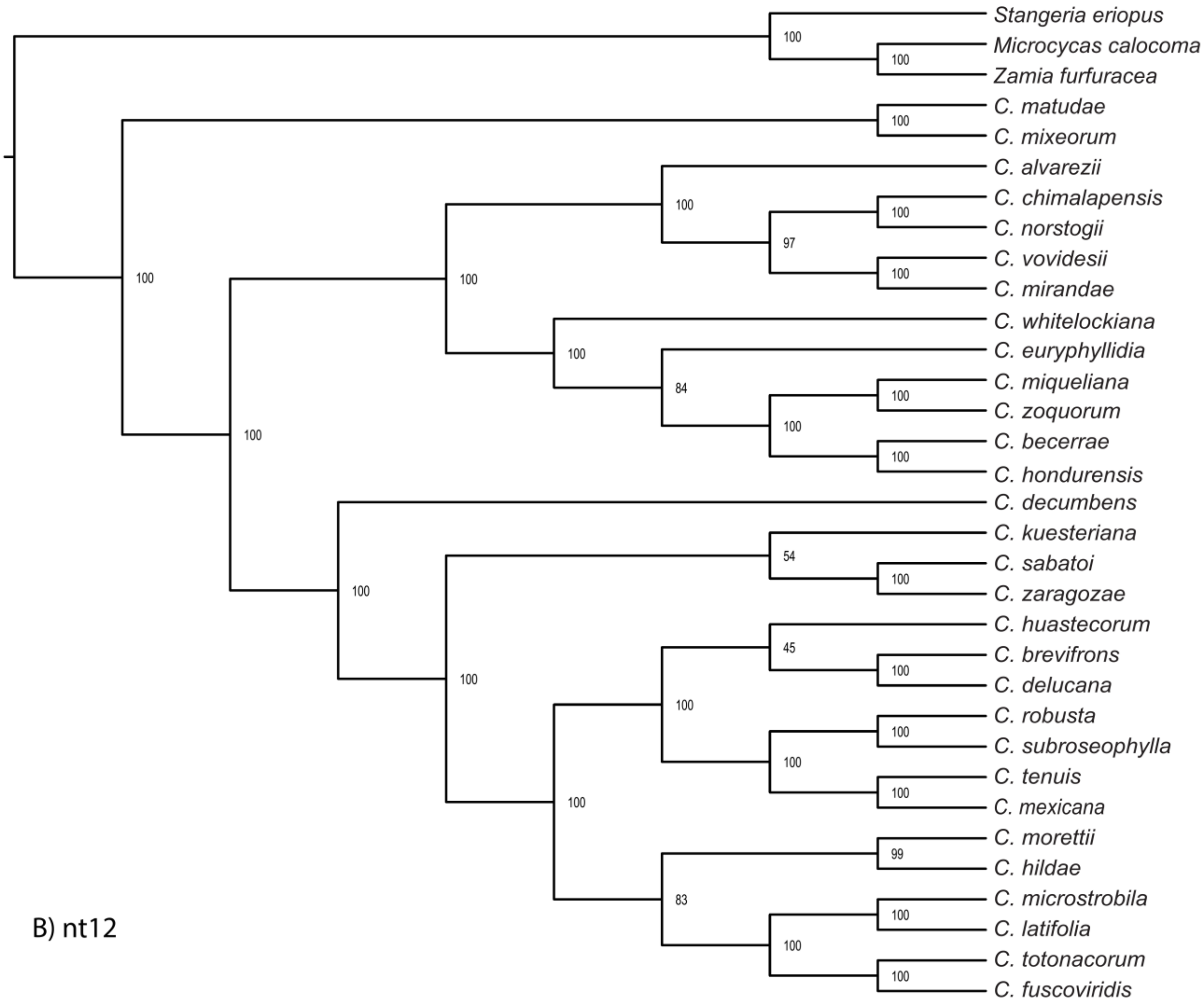

Stangeria eriopus
Microcycas calocoma
Zamia furfuracea
C. matudae
C. mixeorum
C. alvarezii
C. chimalapensis
C. norstogii
C. vovidesii
C. mirandae
C. whitelockiana
C. euryphyllidia
C. miqueliana
C. zoquorum
C. becerrae
C. hondurensis
C. decumbens
C. kuesteriana
C. sabatoi
C. zaragozae
C. huastecorum
C. brevifrons
C. delucana
C. robusta
C. subroseophylla
C. tenuis
C. mexicana
C. morettii
C. hildae
C. microstrobila
C. latifolia
C. totonacorum
C. fuscoviridis
100
100
100
100
97
100
100
100
100
84
100
100
100
100
100
100
54
100
100
45
100
100
100
100
100
99
83
100
100
100

B) nt12

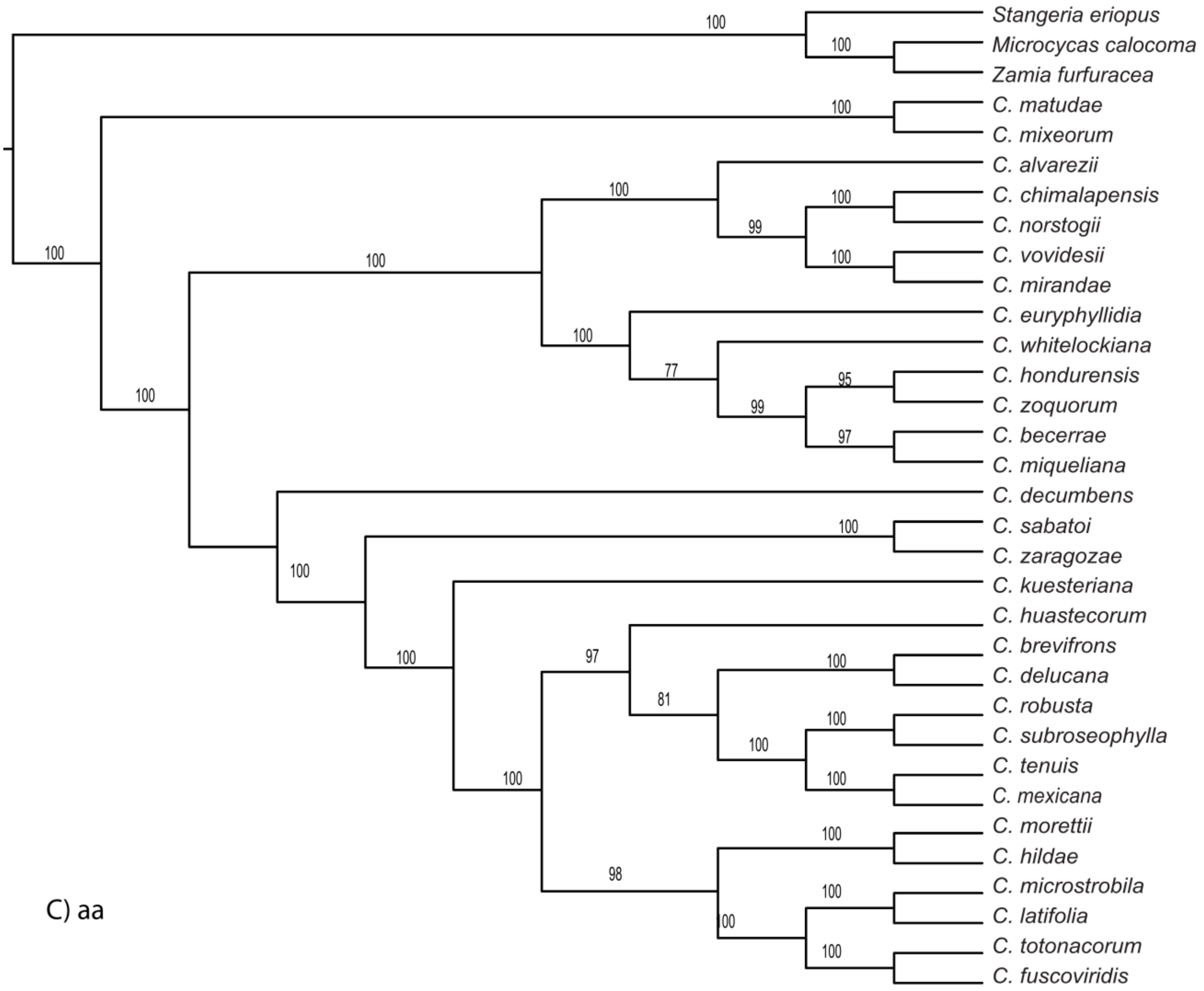

Figure S1: Phylogenetic relationships within *Ceratozamia* based on concatenated dataset of A) nt, B) nt12, C) aa, from 3954 Single Copy Nuclear genes (SCGs).

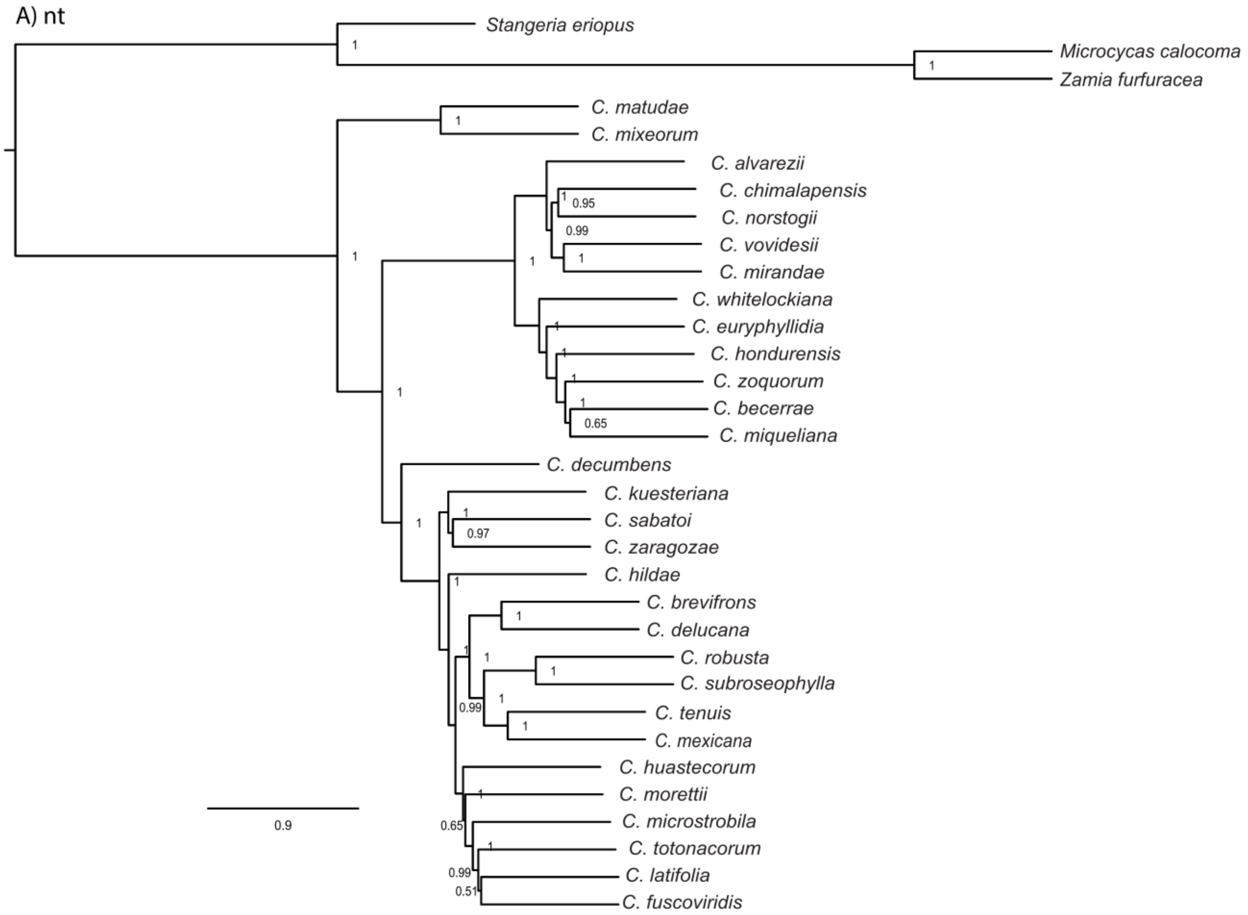
A) nt
Stangeria eriopus
Microcycas calocoma
Zamia furfuracea
C. matudae
C. mixeorum
C. alvarezii
C. chimalapensis
C. norstogii
C. vovidesii
C. mirandae
C. whitelockiana
C. euryphyllidia
C. hondurensis
C. zoquorum
C. becerrae
C. miqueliana
C. decumbens
C. kuesteriana
C. sabatoi
C. zaragozae
C. hildae
C. brevifrons
C. delucana
C. robusta
C. subroseophylla
C. tenuis
C. mexicana
C. huastecorum
C. morettii
C. microstrobila
C. totonacorum
C. latifolia
C. fuscoviridis
0.9

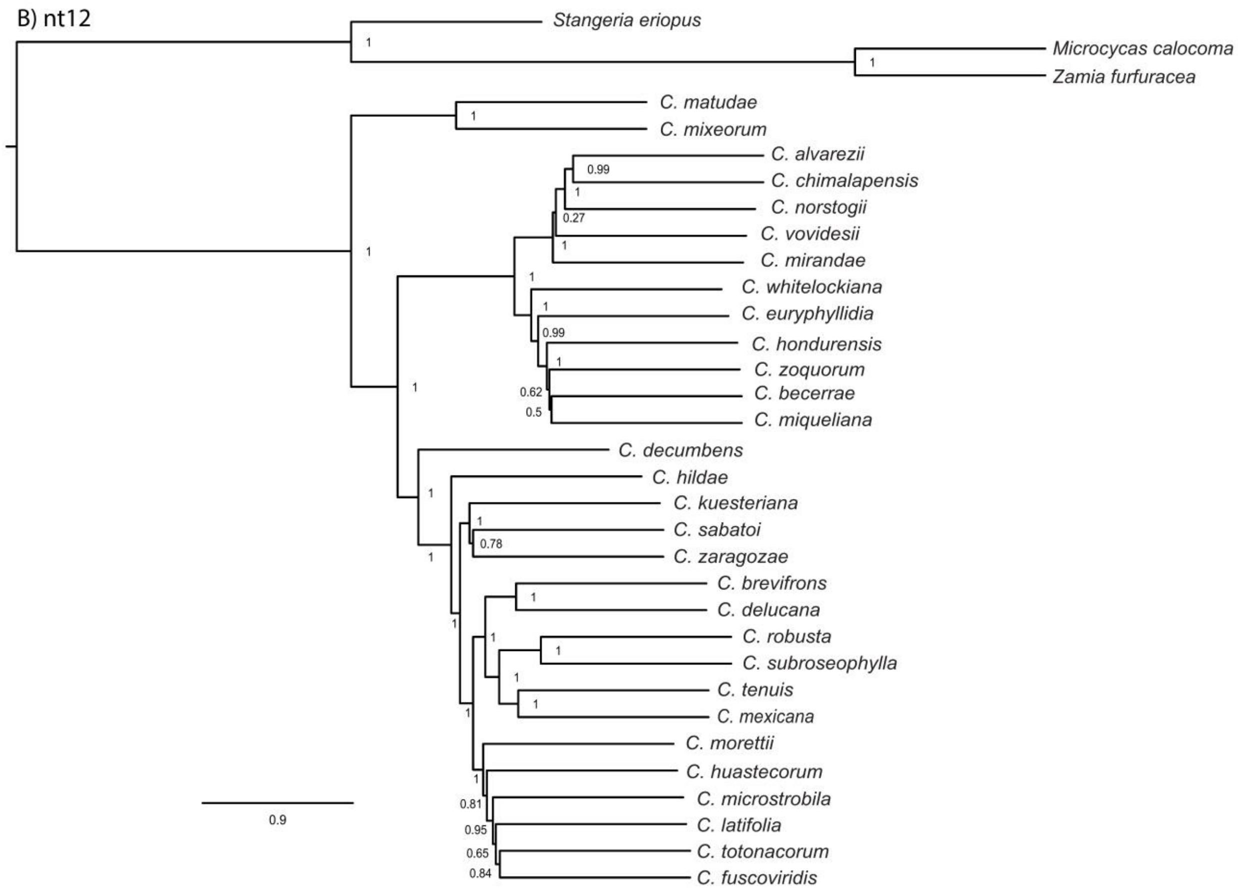
B) nt12
Stangeria eriopus
Microcycas calocoma
Zamia furfuracea
C. matudae
C. mixeorum
C. alvarezii
C. chimalapensis
C. norstogii
C. vovidesii
C. mirandae
C. whitelockiana
C. euryphyllidia
C. hondurensis
C. zoquorum
C. becerrae
C. miqueliana
C. decumbens
C. hildae
C. kuesteriana
C. sabatoi
C. zaragozae
C. brevifrons
C. delucana
C. robusta
C. subroseophylla
C. tenuis
C. mexicana
C. morettii
C. huastecorum
C. microstrobila
C. latifolia
C. totonacorum
C. fuscoviridis
0.99
0.27
0.62
0.5
0.78
0.81
0.95
0.65
0.84
0.9

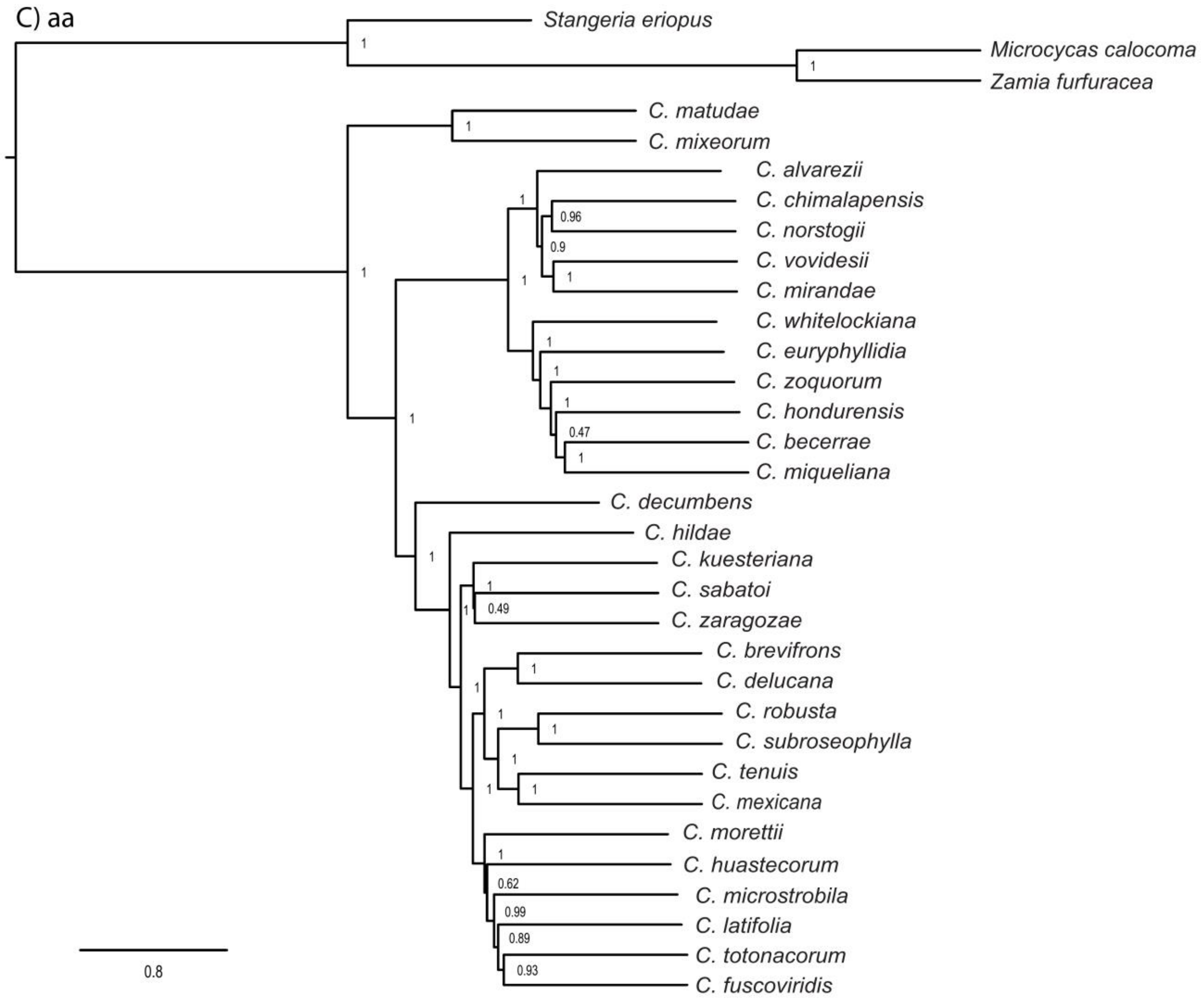

Figure S2: Phylogenetic relationships within *Ceratozamia* based on coalescent dataset of A) nt, B) nt12, C) aa, from 3954 Single Copy Nuclear genes (SCGs).

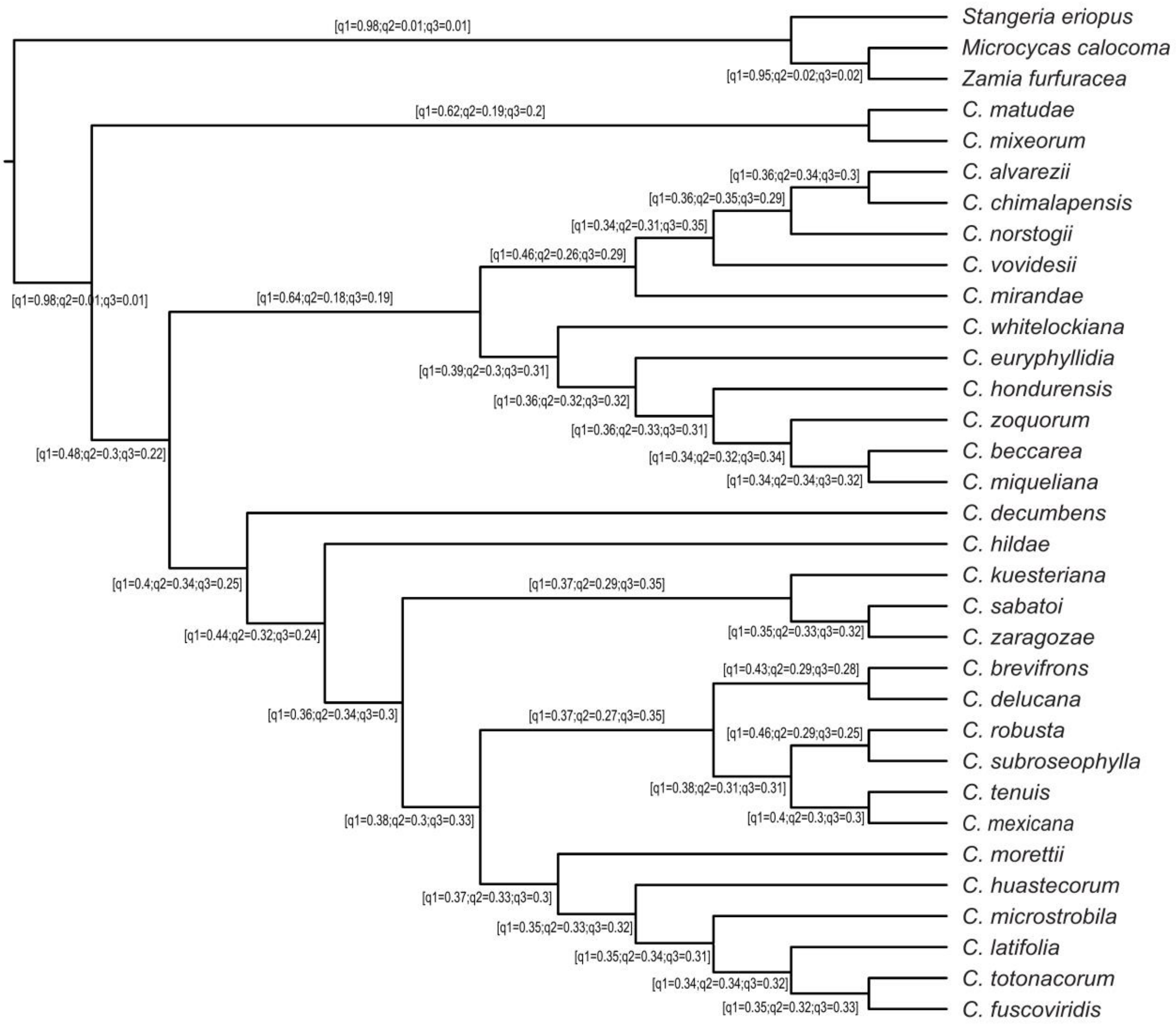

Figure S3: Species trees of the genus *Ceratozamia* generated in ASTRAL-III for nucleotide dataset with 1st and 2nd codon position (nt12). Support values from quartet analysis are mentioned for each node. The low support values of the consensus highlight the amount of incongruence among the gene trees.

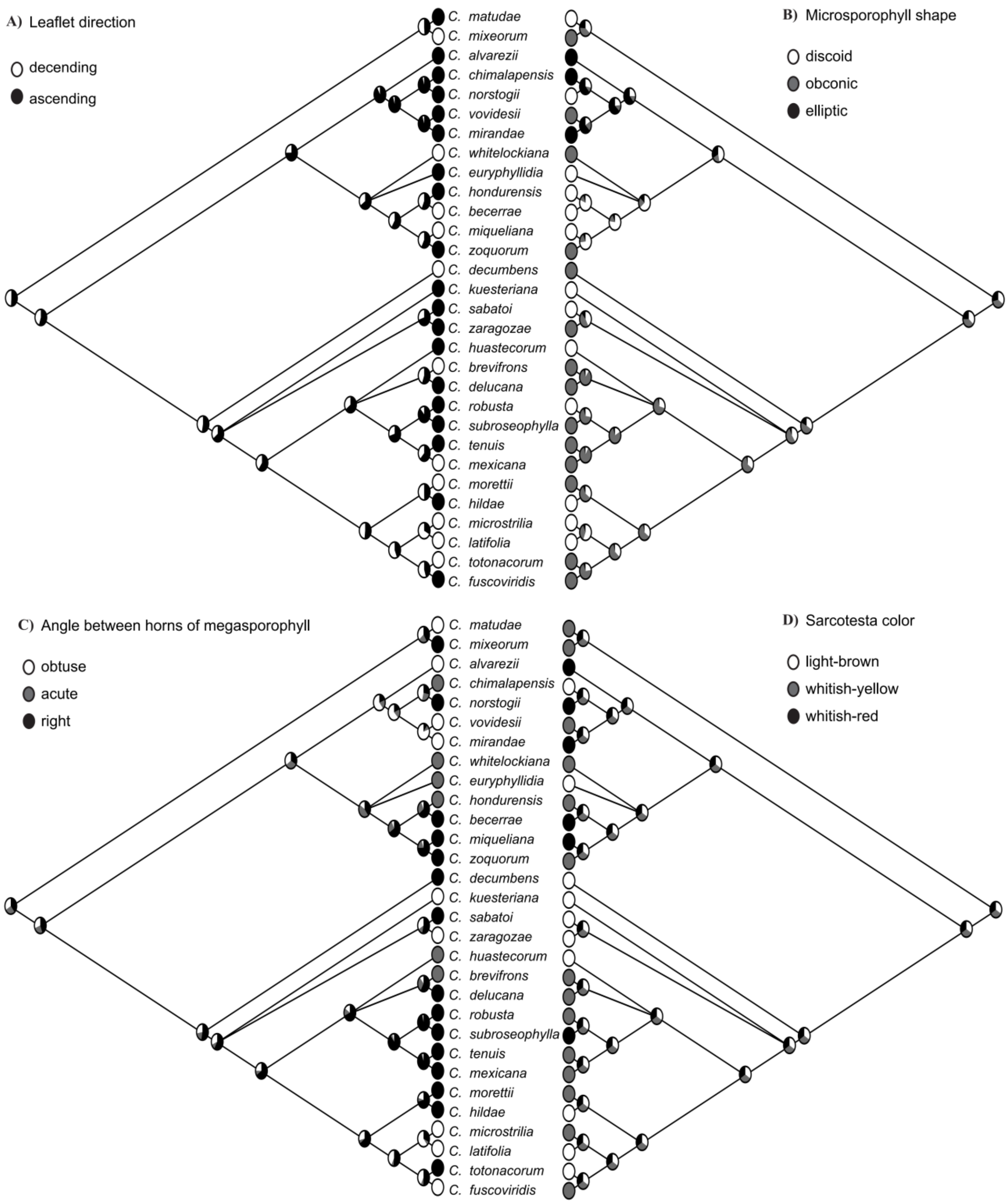

Figure S4: Optimization of morphological characters with complex pattern, based on Mk1 model implemented in Mesquite. (A) Leaflet direction (character 3), (B) Microsporophyll shape (character 6), (C) Angle between horns of megasporophylls (character 10), and (D) Sarcotesta color (character 12) inferred to ML tree of concatenated dataset with all codon position included (nt dataset), with poorly supported branches (BS < 85) collapsed.

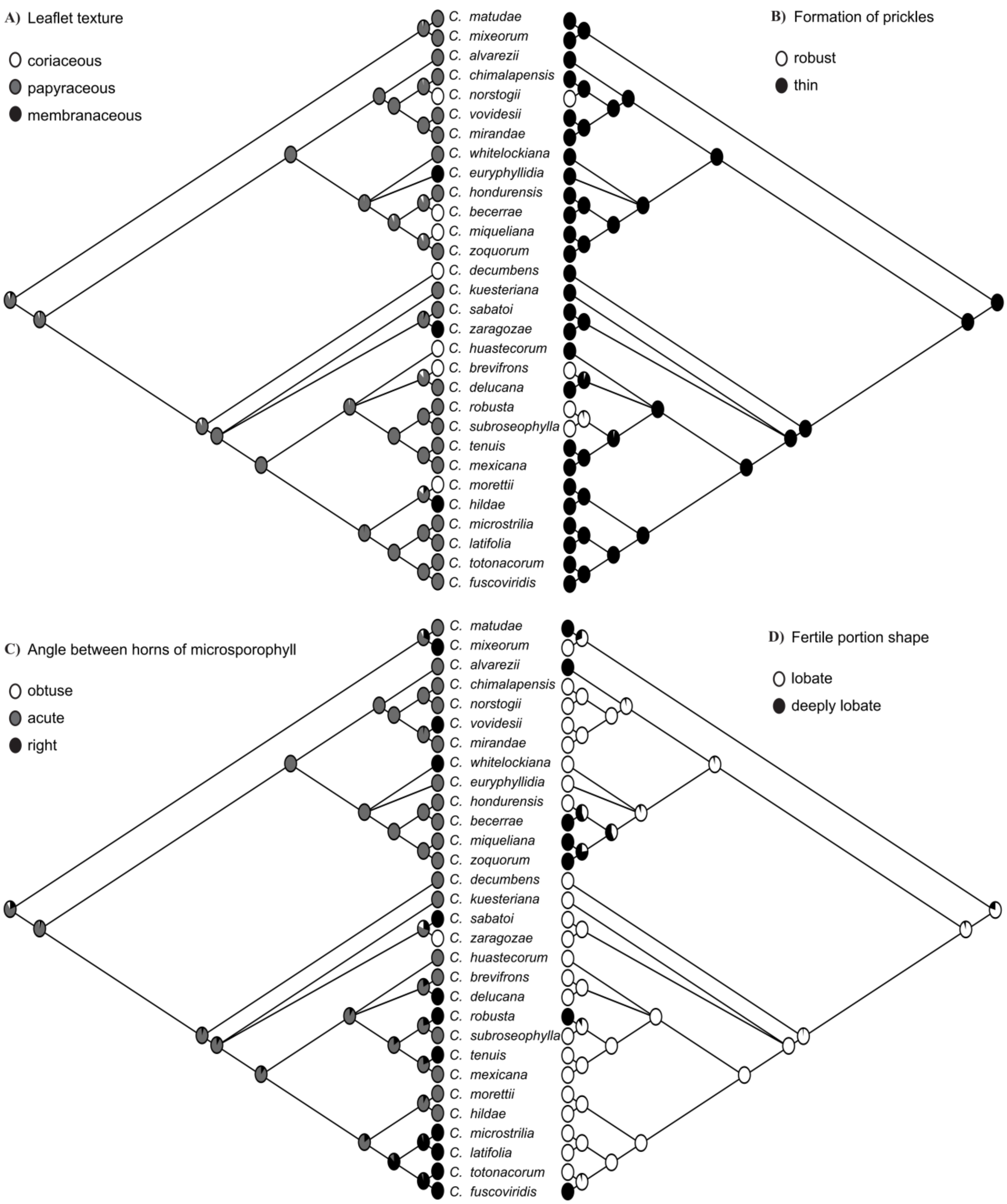

Figure S5: Optimization of less variable morphological characters, based on Mk1 model implemented in Mesquite. (A) Leaflet texture (character 4), (B) Leaflet direction (character 5), (C) Angle between horns of microsporophyll (character 7), and (D) Fertile portion shape (character 9) inferred to ML tree of concatenated dataset with all codon position included (nt dataset), with poorly supported branches (BS < 85) collapsed.
